# Specialism and generalism in social animals in variable environments

**DOI:** 10.1098/rstb.2023.0264

**Published:** 2025-03-20

**Authors:** Koichi Ito, Andrew Higginson

**Affiliations:** ^1^ Faculty of Environmental Earth Science, Hokkaido University, Sapporo, Hokkaido 060-0810, Japan; ^2^ Centre for Research in Animal Behaviour, College of Life and Environmental Sciences, University of Exeter, Exeter EX4 4QG, UK

**Keywords:** specialism, generalism, ants, social insects, environmental variation, cooperation

## Abstract

An important advantage to sociality is division of labour, which is often associated with specialization of group members, such as the polymorphic subcastes of ant workers. Given this advantage, it is puzzling that many social groups do not show clear specialization. Among ants, workers of closely related species have one, two or even three polymorphisms. The degree of specialism of asocial animals depends on environmental variability because specialists will perform poorly in some conditions. Here, we use a numeric model to consider whether the magnitude and type of environmental variability can help to explain the diversity of specialism in cooperative groups. By finding the optimal distribution of group members along a single dimension of specialization for two tasks, we predict when groups should be composed of specialists, generalists, both of these (trimodal) or moderate specialists. Generalism is predicted more when environments are unstable and when task importance—rather than demand—varies but depends on the likelihood that the group can complete all tasks in the range of experienced conditions. The benefit of sociality is strongest in invariable environments and there is selection for redundancy in the workforce, which may explain the widely observed inactivity in social insects.

This article is part of the theme issue ‘Division of labour as key driver of social evolution’.

## 1. Introduction

Social animals that form cooperative groups dominate many ecosystems, and specialization of members for different tasks is common [1–3]. Because reproductive success in social animals is often highly correlated with group-level performance, such specialization is often based on the share of the tasks and resources within the group. Such cooperative task sharing in social animals is known as ‘division of labour’ [4]. Division of labour has evolved in many taxa where related individuals live in groups, such as social amoebae [5], algae [6], sea anemones [7], social insects [8,9], birds [10,11], mammals [12,13] and even viruses [14].

In empirical studies, division of labour is often quantified by the variation in body size as a form of phenotypic polymorphism. The clearest example may be large body size variation within the workers of some species of leaf-cutting ants [15] and fire ants [16,17]. The distribution of body sizes is often continuous and may be unimodal, bi- or even trimodal, and the modes may be specialized to different worker roles [18]. In some species of ant, the distribution of body size differs between colonies or habitats [9,15,19,20] but is often multimodal. In other eusocial animals, such as bumblebees, size variation is striking but unimodal [21]. Different body sizes are for specialization for specific tasks and improve the performance of a particular task to the detriment of the performance of other tasks [18]. Nevertheless, size variation is not always the best indicator for division of labour. For instance, honeybee and bumblebees divide labour according to the age of the workers [22].

Past theories on division of labour have identified several influential factors: food size distribution, number of resource types, group size and resource availability [9,15,19]. A dominant theoretical focus is on the emergence of reproductive specialists, which leads to the evolution of multicellularity, eusociality or cooperative breeding (e.g. [23,24]), but these studies do not consider the variation among non-reproductive units. Oster & Wilson [18] focused on the situation in which a continuous trait value (e.g. body size) determines the handling ability of different sized foods and predicted that the optimal distribution of workers’ traits can be either unimodal or multimodal depending on the distribution of food size, although the maximum number of worker classes is equal to the number of resource types. In other words, the balance between the usability of various food and the cost of producing multiple workers classes determines the emergence of specialists or generalists. Wahl [14], Tannenbaum [25] and D’Orazio & Waite [26] considered cooperation among non-kin that must carry out two tasks and showed that the coexistence of specialists and generalists can occur, depending on group size and the relative performance of specialists compared with generalists. In these cases, the advantage of generalists compared with the specialists is that generalists can flexibly switch tasks depending on the types and tasks of other group members.

Variability in environmental conditions, on the other hand, has not been considered despite increasing awareness of its importance in the behaviour of cooperating groups (e.g. [27]). Organisms usually face temporal–spatial variability in environmental conditions [28–30]. In asocial animals, adaptive strategies for coping with variable environments can divided into two types: specialize for a limited range of conditions or generalize for a wide range of conditions with the cost of lower maximum performance. The evolution of specialists and generalists has been widely observed in various taxonomic groups [31] and has been theoretically investigated [28,32–34]. Previous theoretical studies predicted that adaptative strategies for variable environments should be influenced by the particulars of the trade-off between the adaptations for different environments and the statistical patterns of environmental fluctuation (e.g. spatial/temporal variation or interval of the fluctuation [34]). In sum, past theory on other types of biological adaptation has predicted that generalism is selected when the trade-off between the performance in different conditions has a convex shape or the environmental variation is temporal rather than spatial, yet it is not clear whether the condition holds for the evolution of specialization in social species.

Here, we focus on the influence of environmental variability on specialism in a social group. Environmental variation might alter the group-level task demand, task processing efficiency or its importance to reproductive success, but a group consisting of generalists can adapt to such changes of task conditions. Unlike asocial animals, the adaptation for a stochastic environment in social animals will be more complex because each individual can be generalist or specialist. In other words, the allocation of specialism within the group should be considered to understand the emergence of individual variation, such as worker ant size polymorphism, in the fluctuating environments in which most species have evolved. To our knowledge, there is no theoretical framework for understanding the influence of environmental variability on the allocation of specialism within groups.

We used a simulation approach to predict the optimal allocation of specialists and generalists within a colony of eusocial animals as a function of environmental variability. In order to establish a theoretical foundation for understanding these influences, we considered the most simple possible situation: we focus on a colony of eusocial animals (so that all members in the colony share the reproductive success) and assume that the colony must do two tasks (e.g. collect two types of food items) and there is a trade-off between each individual’s abilities to do the two tasks. Rather than limiting each individual’s options to extreme specialism or absolute generalism, we allow degrees of intermediate specialism. We assume that the fitness of the colony is inversely proportional to the total amount of uncompleted tasks averaged across all possible conditions. We modelled environmental variability as a frequency distribution of the required amount of the two tasks and predicted the frequency distribution of individual specialism as that which maximizes colony fitness.

## 2. The model

### (a) Tasks and pay-off of the colony

Consider a colony of eusocial animals. There are two tasks that the colony must carry out, task A and task B. There is a trade-off between the ability at the two tasks at the individual level: a specialist in either task A or B is poor at doing the other task (B or A) compared with a generalist. We assume that the colony can produce *m* classes of workers that have different abilities. The abilities at task A and task B of the *i*-th class, *a_A_*_,*i*_ and *a_B_*_,*i*_, are determined by the power functions


(2.1a) $$a_{A,i}={\left(\frac{m-i}{m-1}\right)}^{\alpha }\;and$$

(2.1b) $$a_{B,i}={\left(\frac{i-1}{m-1}\right)}^{\alpha }.$$

**Figure 1.**
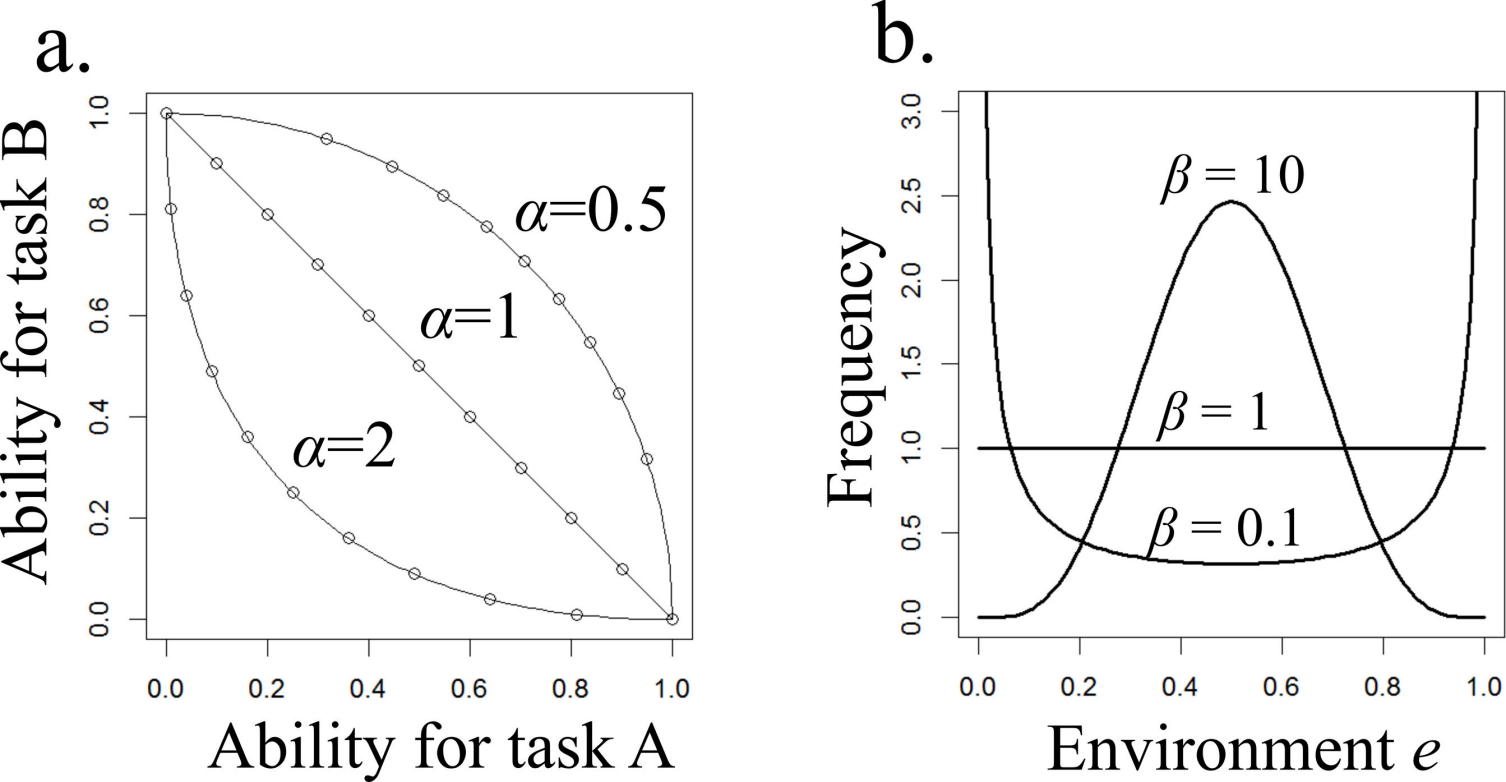
(a) The abilities of individuals when doing task A and task B for *m* = 11 and three values of the parameter *α*. (b) The influence of environmental invariability *β* on the frequency distribution of the environmental states *f*(*e*). If *β* is infinitely large, *e* is always 0.5, and when *β* is zero, *e* is 0 or 1 each with a probability of 0.5; we consider intermediate values 0 > *β* < ∞.

These equations imply that the first class (*i* = 1) is a task A extreme specialist that is unable to do task B ($a_{A,1}=1,a_{B,1}=0$), and the *m*-th class (*i* = *m*) is a task B extreme specialist that is unable to do task A ($a_{A,m}=0,a_{B,m}=1$). Intermediate classes individuals 1 < *i* < m can do both task A and task B, although their ability is lower than the specialist of each task. The exponent *α* determines the shape of the trade-off between task abilities (figure 1a). We also assume this trade-off is symmetrical: the exponent *α* has the same value in both of equations (2.1a) and (2.1b). Note that we assume that each worker does one task at any given moment even if it can do both tasks.

The strategy of the colony is the proportion of workers in each class. Consider a colony where the proportion of workers belonging to *i*-th class is *x_i_* (i.e. $\sum_{i=1}^{m}x_{i}=1$). At each step, the colony tries to complete two tasks with task demand, *d*_A_(*e*) and *d*_B_(*e*), which may vary depending on the condition of the environment *e*. Each worker within the colony is allocated to either of two tasks depending on the demand. When the proportion of the *i*-th class workers allocated to task A is *p_i_*, the amount of uncompleted task A and task B can be represented as


(2.2a) $$\max_{x,p}\left(0,d_{A}\left(e\right)-k_{A}\left(e\right)\sum_{i=1}^{m}a_{A,i}p_{i}x_{i}\right)\;and$$

(2.2b) $$\max_{x,p}\left(0,d_{B}\left(e\right)-k_{B}\left(e\right)\sum_{i=1}^{m}a_{B,i}\left(1-p_{i}\right)x_{i}\right),$$

respectively, where $a_{A,i}p_{i}x_{i}$ and $a_{B,i}(1-p_{i})x_{i}$ are the classes’s effort on task A and task B. *k*_A_(*e*) and *k*_B_(*e*) determine the overall efficiency of doing task A and task B per unit effort in environmental condition *e*. We assumed that the pay-off of the colony *w* is the negative value of the sum of the uncompleted tasks, i.e.


(2.3a) $$w\left(x,p,e\right)=w_{A}\left(x,p,e\right)+w_{B}\left(x,p,e\right)$$

where


(2.3b) $$w_{A}\left(x,p,e\right)=-\theta_{A}\left(e\right)\max\left(0,d_{A}\left(e\right)-k_{A}\left(e\right)\sum_{i=1}^{m}a_{A,i}p_{i}x_{i}\right)\;\;\;\;and$$

(2.3c) $$w_{B}\left(x,p,e\right)=-\theta_{B}\left(e\right)\max\left(0,d_{B}\left(e\right)-k_{B}\left(e\right)\sum_{i=1}^{m}a_{B,i}\left(1-p_{i}\right)x_{i}\right),$$

and *θ_A_(e*) and *θ*_B_(*e*) are the relative importance of the tasks for colony fitness in environmental condition *e*.

Although there is discussion about whether social animals can optimize the allocation of workers across tasks [35,36], for our purposes, we assumed that the colony can realize the optimal allocation of available workers into the two tasks: $p^{*}=\left\{p_{1}^{*},p_{2}^{*}\ldots p_{m}^{*}\right\}$ which maximizes the colony pay-off for given x and *e* (see electronic supplementary material, appendix A for the methodological details). By using this optimal allocation, we can rewrite the minimum fitness cost to the colony of uncompleted task as w^*^(***x***, *e*) = *w*(***x***, ***p***, *e*).

We assume that the focal colony repeatedly completes this task allocation process and obtain pay-offs for different environmental conditions. For instance, on a daily basis, the colony may respond to different demands for the two tasks by allocating workers appropriately. The reproductive productivity (i.e. total fitness) of the colony *W* is the mean value of the pay-offs, i.e.,


(2.4) $$\;W\left(x\right)=\int_{E}^{\;}f\left(e\right)w^{*}\left(x,e\right)\;\;\;de$$

where *E* is the possible range of the environment *e* and *f* is the probability density function of the environment *e*. By using numerical simulation, we investigated the optimal proportion of the worker classes within the colony x^*^ that maximizes *W*.

### (b) Three scenarios of environmental variability

Environmental variability distribution *f*(*e*) alters the best combination of abilities, which will influence the optimal distribution of specialism in the colony x*. We assumed that the environmental condition is represented as a continuous value 0≤e≤1, and the frequency follows a beta distribution, i.e.


(2.5) $$f\left(e\right)=\frac{e^{\beta -1}{\left(1-e\right)}^{\beta -1}}{B\left(\beta ,\beta \right)}$$

where *B* is the beta function. Parameter *β* determines the pattern of variability. When *β* > 1, this probability distribution is hill shaped, and the environmental value *e* is mostly around 0.5, such as in an invariable environment (figure 1b). On the other hand, when 0 < *β* < 1, the distribution is valley shaped, so that the environmental value *e* tends to be either zero or one at any given time, implying variable environmental conditions. Because the fluctuation of the environmental value is smaller when *β* is larger, hereafter we refer to *β* as ‘environmental invariability’.

The environmental value *e* potentially affects three pairs of task parameters: task demand ($d_{A},d_{B}$), task efficiency ($k_{A},k_{B}$) and task importance ($\theta_{A},\theta_{B}$). Depending on what the focal tasks are (e.g. food collection, caring for larvae, nest maintenance or colony defence) and what is the focal environmental condition (e.g. weather, temperature or food/predator density), the influence of environmental value *e* on the three pairs of task parameters will be different. In order to consider the various types, we considered the following three scenarios.

#### (i) Scenario 1

**Figure 2.**
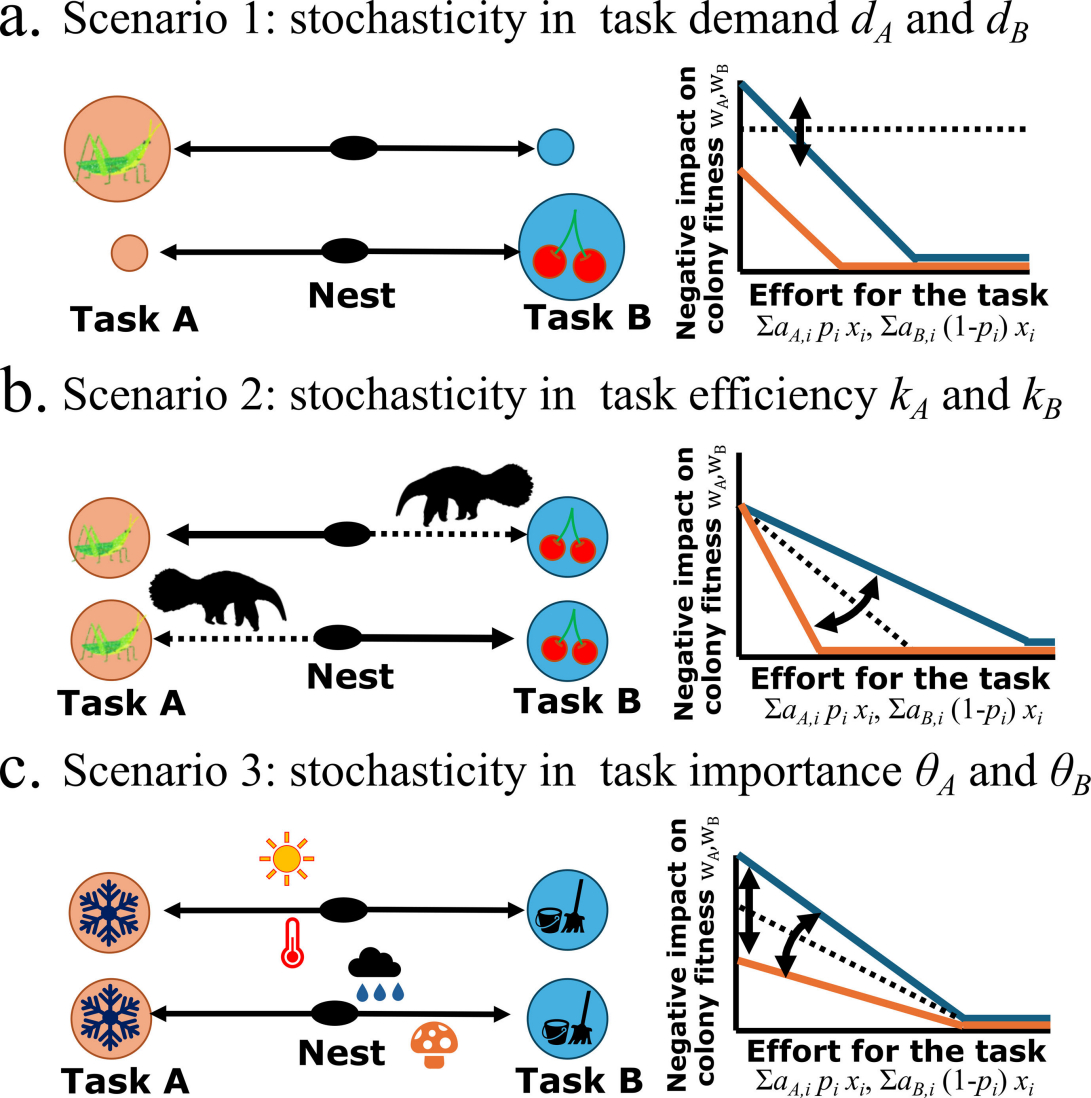
Visualization of the three scenarios of environmental variation showing (left) cartoon and (right) the effect of the effort dedicated to a task on the negative impact on the colony fitness. (a) When the availability of two types of food resources fluctuates, the task demand might fluctuate, so the intercept varies. (b) When the accessibility to two types of resources that are located in far places from the nest is changed by the predators, the efficiency for the task process might fluctuate, so the slope varies. (c) Depending on the weather, the risk of nest overheating and spread of fungi might fluctuate, which varies the importance of nest cooling tasks and cleaning tasks on colony fitness. This is equivalent to both the intercept and slope varying.

First, we considered the scenario that the ratio of task demand, i.e. the ratio of *d_A_* to *d_B_* fluctuates due to environmental variability, while the total task demand *d*_A_+ *d*_B_ is a constant value d, i.e.


(2.6a) $$d_{A}\left(e\right)=de$$

(2.6b) $$d_{B}\left(e\right)=d\left(1-e\right)$$

while other parameters are constant (*k*_A_ = *k*_B_ = *k*/2, *θ*_A_ = *θ*_B_ = *θ*/2). This might represent a situation that the availability of two types of food resources changes depending on the environment, while the total demand of food by the larva is constant (figure 2a).

#### (ii) Scenario 2

In the second scenario, we considered the fluctuation of the ratio of task efficiency ($k_{A},k_{B}$), i.e.


(2.7a) $$k_{A}\left(e\right)=ke$$

(2.7b) $$k_{B}\left(e\right)=k\left(1-e\right)$$

while other parameters are constant (*d*_A_ = *d*_B_ = *d*/2, *θ*_A_ = *θ*_B_ = *θ*/2). This scenario might correspond to a situation in which two types of resource are collected from distant places. The availability of the access route for those resources might be affected by the location of predators or obstacles, which will alter task efficiency because of the time taken by vigilance or detours (figure 2b).

#### (iii) Scenario 3

Last, we considered the fluctuation of the ratio of task importance (*θ*_A_, *θ*_B_), i.e.


(2.8a) $$\theta_{A}\left(e\right)=\theta e$$

(2.8b) $$\theta_{B}\left(e\right)=\theta \left(1-e\right)$$

while other parameters are constant (*d*_A_ = *d*_B_ = *d*/2, *k*_A_ = *k*_B_ = *k*/2). This scenario might correspond to a situation that a cleaning task to prevent the spread of fungi has more importance to colony reproductive success on a wet day, while the nest-cooling task has more importance to colony reproductive success on a dry day (figure 2c). Importantly, in both cases, the amount of task demand is constant, but the negative influence on the reproductive success by the uncompleted tasks (i.e. spread of fungi or overheat of the colony) can fluctuate.

Note that in these three scenarios, the parameters of two tasks are correlated. Another possible assumption is that the parameters of two tasks fluctuate independently. However, such an assumption causes very extreme situations, such as the demands of both tasks are too high or the efficiency of both tasks is very low (since the beta distribution is adopted for representing the environmental variability, such situations can occur with non-negligible probability). In this study, we focused on the role of generalists within the colony under environmental variability, and such extreme scenarios are not helpful for our purpose.

### (c) Optimal strategy of asocial animals

To investigate the fitness advantage of sociality, we also predicted the optimal strategy of asocial animals. Although the model framework is mostly the same as the social animal case, the asocial animal can allocate to only one class of the specialisms (i.e. *x_i_* = 1 and *x_j≠i_* = 0) because asocial animals must complete tasks alone. Under this limitation, we predict the best strategy for an asocial animal.

## 3. Results

**Figure 3.**
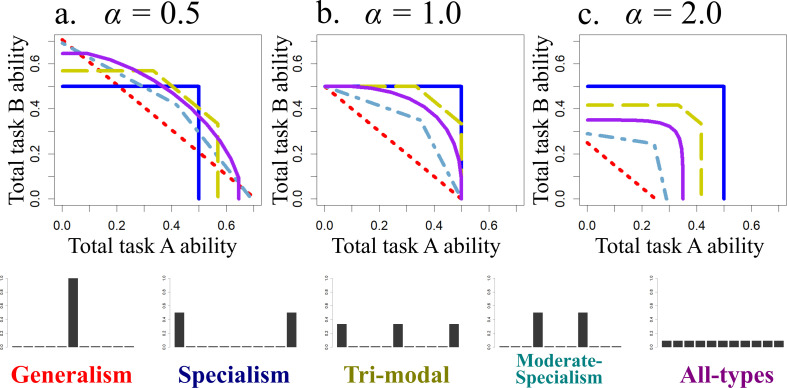
The combinations of the total ability for tasks A and B (upper panels) of the typical five examples of distribution of specialisms (lower panels). The five typical examples of the distribution of specialisms are *generalism* (only generalists; *x*_6_ = 1, red dotted), *specialists* (pair of extreme specialists for task A and B; *x*_1_ = *x*_11_ = 0.5, blue solid), *trimodal* (both extreme specialists and generalists; *x*_1_ = *x*_6_ = *x*_11_ = 1/3, yellow dashed), *moderate-specialists* (pair of moderately specialized classes; *x*_4_ = *x*_8_ = 0.5, cyan dot-dash) and *all-types* (all classes exist equally, *x_i_* = 1/11, purple solid). Upper panels show the maximum combination of the total ability for task A and B for each typical example. The colony with each distribution of specialisms can perform the amount of tasks underneath each curve.

When *α* ≥ 1 (trade-off is not concave), regardless of the parameter values or scenario, the optimal distribution of specialism is always complete specialism, i.e. 50% of workers are task A extreme specialists and 50% of workers are task B extreme specialists (i.e. *p*_1_ = *p_m_* = 0.5). This is because when *α* ≥ 1 the ability of generalists is so low that the total ability of two generalists is smaller than a specialist. In such a case, a pair of specialists for both tasks always has better task overall performance than the two generalists (i.e. figure 3b,c).

When *α* < 1, on the other hand, various types of distribution of specialism are optimal depending on the parameter values and scenarios. This occurs because generalists can complete tasks outside of the range that specialists can manage (figure 3a, cf. red dotted to blue solid lines), and having some moderate specialists can do better still for some task demands (cf. other lines to red dotted lines).

**Figure 4.**
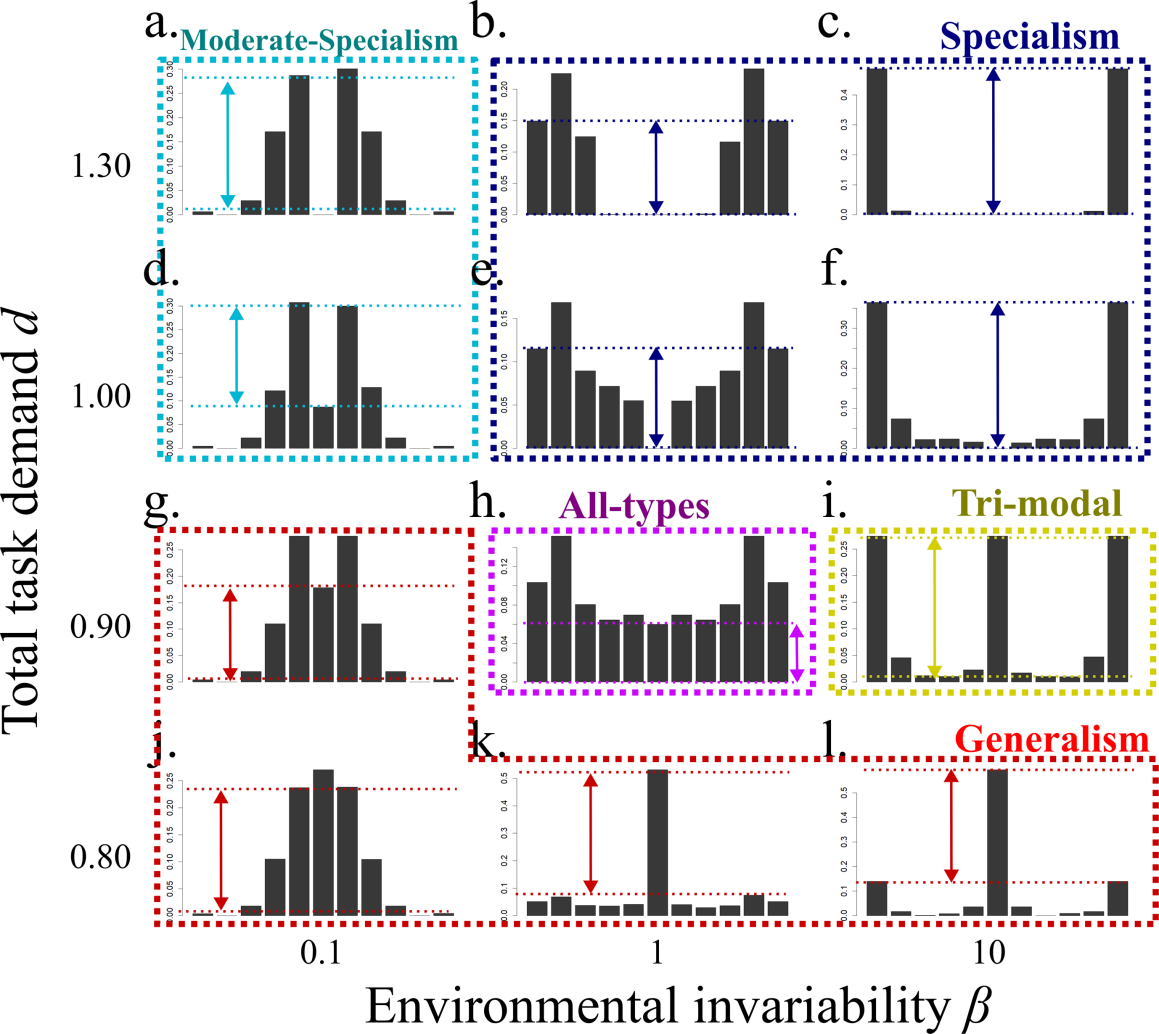
The optimal distribution of specialism in the scenario of variability in task demand. In each panel, bars at the extreme left and extreme right show the proportion of specialists for task A and task B, respectively, and the centre is that of generalists. Arrows between two dotted lines show the range of the majority category of the distribution type. Values of *d* and *β* are shown at the end of the rows and columns, respectively. Other parameter values: *m* = 11, *α* = 0.5, *k* = 2, *θ* = 2.

Figure 4 shows the optimal distribution of specialisms in scenario 1 for four values of the total task demand *d* (rows) and three values of environmental invariability *β* (columns). Following the method described in electronic supplementary material, appendix B, we automatically categorized the optimal distribution into five categories of distribution (i.e. specialism, generalism, trimodal, moderate-specialism and all-types). The group may consist of only specialists for task A and task B (figure 4b,c,e,f, ‘specialism’), or the group may have both specialists and generalists (figure 4i, ‘trimodal’). In other conditions, the distribution of the proportion is unimodal and most are generalists (figure 4j–l, ‘generalism’), or there may be no absolute generalists and the distribution bimodal, so that most individuals are moderate-specialists for task A and task B (figure 4a,d,g, *‘*moderate-specialism’). Finally, the distribution may have all classes in non-negligible proportions (figure 4h, ‘all-types’).

### (a) Scenario 1: variability in task demand determines the extent of specialism

**Figure 5.**
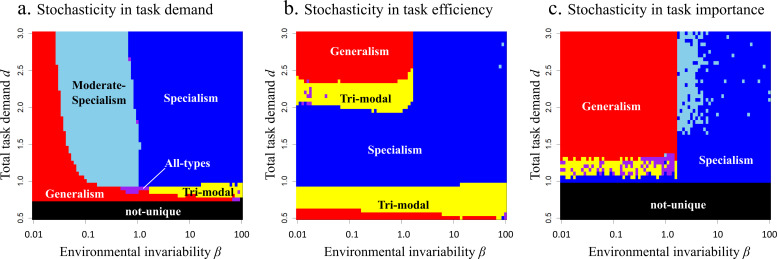
The optimal specialism types for the total task demand *d* and the environmental invariability *β* in three scenarios: stochasticity in (a) task demand, (b) task efficiency and (c) task importance. Colours indicate: red: generalism (e.g. figure 4j–l); blue: specialism (e.g. figure 4b,c,e,f); yellow: trimodal, i.e. specialists and generalists (e.g. figure 4i); cyan: moderate-specialism (e.g. figure 4a,d,g); purple: all-types (e.g. figure 4h); black: optimal distribution cannot be uniquely defined. Other parameter values: *m* = 11, *α* = 0.5, *k* = 2, *θ* = 2.

Figure 5a shows the categorization of the optimal distribution when there is variation in task demand (scenario 1) for a range of values of the total task demand *d* (vertical axes) and the environmental invariability *β* (horizontal axes). When the total task demand is large (*d* > 0.7), the optimal distribution type is specialism (blue area) in invariable environments (*β* > 1). This is because when the demand of two tasks is similar level, specialism has the highest ability (only the blue curve reaches (0.5, 0.5) in figure 3a). On the other hand, when the environment is very variable generalism is optimal (red area in figure 5a), because generalism has the highest performance when there is only demand of either task (only the red curve reaches to (0.0, 0.7) and (0.7 0.0) in figure 3a). Generalism is also optimal when the total task demand is small (*d* < 0.7) because in this case tasks can be mostly completed even by the generalists. Trimodal and moderate-specialism (yellow and sky-blue areas) emerges between the parameter region of specialists and generalists, but trimodal is the optimal distribution in invariable environments (*β* > 1), while moderate-specialism is optimal in variable environments (*β* < 1). This is because of the qualitative difference between these two types. Trimodal has good performance under the certain range of the bias between the amount of two task by allocation of generalists, but after all generalists are allocated to either task, there is no further flexibility in task performance (yellow curve in figure 3a, see also electronic supplementary material, figure C1a). On the other hand, moderate-specialism does not have high enough performance especially when both task abilities are required, but it can maintain enough flexibility even when there is only either task (sky-blue curve in figure 3, see also electronic supplementary material, figure C1b). Because of this difference, in invariable environments trimodal is optimal because the colony needs only a limited range of flexibility, while in variable environments moderate-specialisms is optimal because the colony needs to be more flexible.

### (b) Scenario 2: there is an effect of variability in task efficiency only when task demand is high

Figure 5b shows the result of stochasticity in task efficiency (scenario 2). When the environmental invariability is high (*β* > 1) , the outcome is similar to the scenario 1 predictions (i.e. for small total task demand, generalism is optimal because mostly all tasks can be completed even by the generalists; at the moderate task demand, trimodal is optimal because the colony needs more performance than generalists can manage, but the required flexibility is limited in an invariable environment, and at high task demand, specialism is optimal due to their high task performance in invariable environments).

When the environment invariability is low (*β* < 1), on the other hand, the predictions change (cf. left of figure 5a,b). This is because the processing efficiency of the two tasks will often be extremely different, so that the optimal allocation of workers will depend on the total task amount *d*. When the total task demand is very high, it is better to ignore inefficient tasks and just allocate all workers on the more efficient task; then generalists are preferable due to their high flexibility. If the total task demand is moderate, on the other hand, the more efficient task can be completed even by a half of specialists in the colony. Then, the task processing ability of inefficient task is more important for colony-level fitness than flexibility, so specialists are better preferable due to their high task performance (note that in this scenario there is a constant amount of task demand regardless of the environment; see figure 2b). When the task demand is very low, again generalism is optimal more efficient as tasks can be completed easily and the inefficient task can be also mostly completed; then high flexibility of generalism becomes a large advantage for processing all tasks. Between the parameter area of specialism and generalism, trimodal is optimal. This is because, as we described in above, the advantage of generalists is the flexible allocation to more efficient tasks so that the required range of flexibility is limited.

### (c) Scenario 3: the effect of task demand is smaller when task importance varies

Figure 5c shows the result of stochasticity in task importance (scenario 3). Again, specialism is optimal when the environmental invariability is high (*β* > 1) and generalism is optimal when the environmental invariability is low (*β* < 1). Interestingly, moderate-specialism is predicted when the environmental invariability is moderate (*β* is around 1) and total task demand is high. Under high total task demand, tasks cannot be completed even if the colony allocates the worker on either task. Then, the biased allocation to the more important task is preferable and then moderate-specialism is the optimal distribution due to their higher performance when the allocation is biased to one task (figure 3a; electronic supplementary material, figure C1). On the other hand, when the total task demand is low and environment variable, trimodal is optimal, because the task demand is sufficiently low that both tasks can be mostly completed; then limited flexibility is enough for complete all of the tasks.

### (d) Effects of increasing the ability of generalists strongly depend on the scenario

When the trade-off between two tasks (*α*) is less severe (small *α*), the specialism region becomes narrower regardless of the scenario or task demands (electronic supplementary material, figure C2), but even then, specialism is the best allocation when environmental invariability is very high (*β* ≫ 1). When task demand is high and there is variability (electronic supplementary material, figure C2a,d,g) *α* has little effect but when task demand is low the high ability of generalists (small α) leads to a lack of clear classes. When task demand is low and task efficiency (electronic supplementary material, figure C2e,h) or task importance (electronic supplementary material, figure C2f,i) varies, small *α* results in trimodal distributions.

Increasing the baseline task efficiency *k* has the same influence as a reduction of total task demand *d*, and change of the baseline task importance *θ* has no influence on the optimal strategy (see electronic supplementary material, appendix D for the mathematical proof).

Throughout we have assumed that the effect of effort on the remaining tasks is linear until it reaches zero (figure 2). In reality, effort on many tasks will have diminishing returns, such as when prey availability is reduced by foraging. In electronic supplementary material, appendix E, we show the effect on the predictions of relaxing this assumption to capture diminishing returns and conclude that the results and insights are broadly unaffected.

### (e) The advantage of sociality is strongest in invariable environments when task demand is low

**Figure 6.**
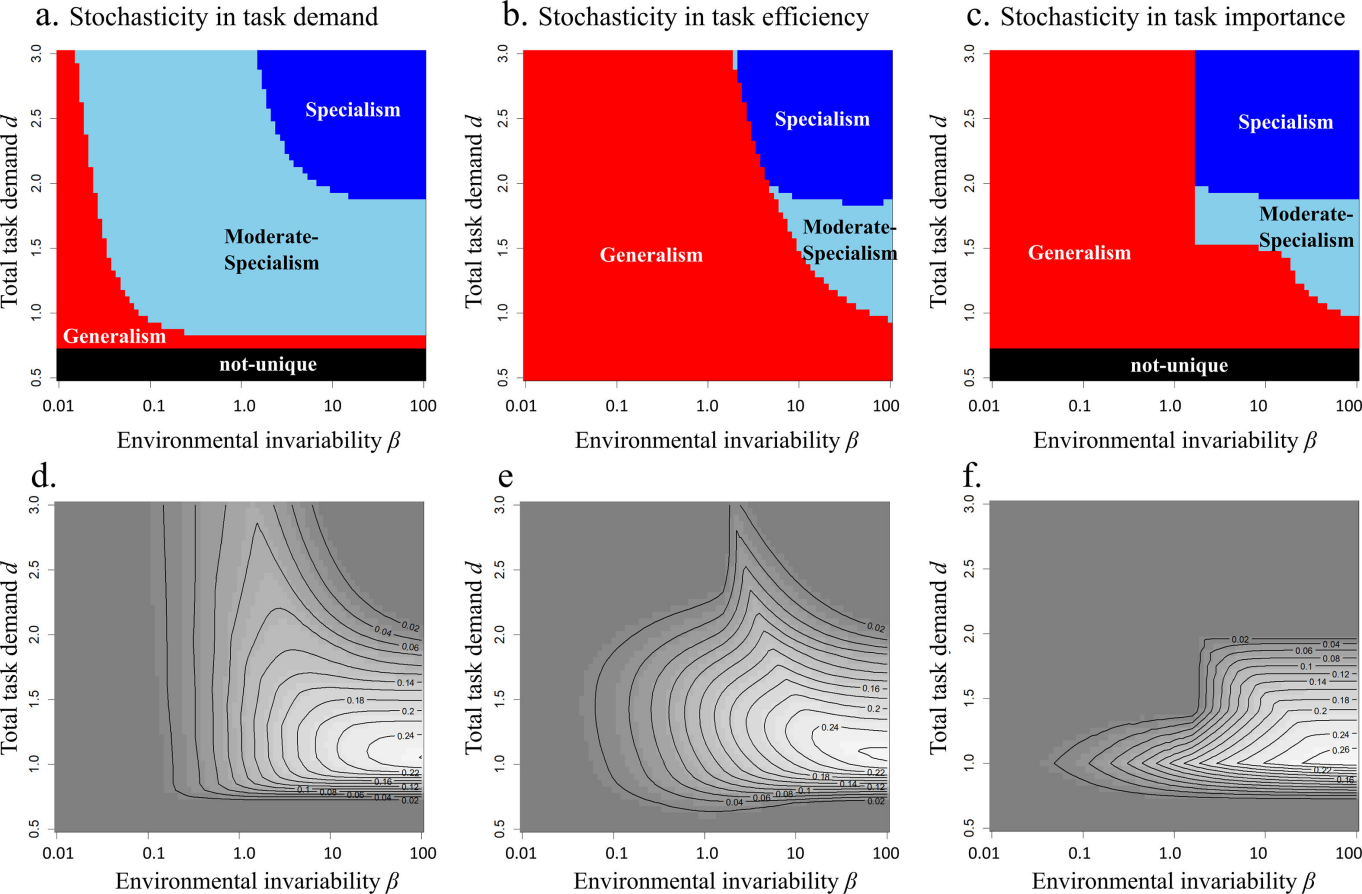
The optimal specialism type of asocial animals (upper panels) and the fitness advantage of social animals over asocial animals (lower panels). Note that the meaning of specialism and moderate-specialism is different (i.e. there is only a task A specialist or task B specialist even in specialism and moderate-specialism). Other parameter values are as in figure 5.

The optimal strategy for asocial animals (figure 6a–c) is somewhat similar to social animals with some interesting differences. Overall, generalism and moderate specialism are much more common, with the greatest difference for moderate demand (1 < *d* < 2) in invariable environments where social animals tend to be specialist. Social animals always have higher fitness than asocial ones, because the possible strategy of asocial animals is a special case (subset) of that of social animals, i.e. asocial animals must allocate to a single class. The fitness advantage of sociality over asocial animals strongly depends on environmental fluctuation (figure 6d–f), with little advantage in variable environments when both social and asocial animals are generalists, but in invariable environments a mixture of specialists performs much better than a moderate specialist. The advantage of sociality is stronger when they usually complete all tasks (bottom-right) such that often individuals will have no tasks to perform, suggesting that there is selection for redundancy in the workforce. The fitness advantage of sociality also disappears when the total task demand *d* is sufficiently large (i.e. *d* > 2), because in these areas task A (or B) is rarely completed even by an specialist, and the division of labour does not greatly reduce the total amount of remaining tasks.

## 4. Discussion

Previous studies of specialism under the influence of environmental variability have mostly focused on adaptation at the individual scale, assuming that the mean performance of each asocial animal determines the degree of specialism [34]. In social animals, however, reproductive success depends on group-level performance. Selection on social animals from environmental variation can therefore involve a distribution of specialization within the group. Here, we have assumed that individuals can be specialized to one of the two tasks along a continuum, which is an advance from previous studies that typically assumed only that individuals can be either (extreme) specialists or generalists. This increased sensitivity enables to us to make a range of predictions about the distribution of specialism in groups and predict four broad categories to enable our predictions to be tested to acquire general insights about the continuous distribution of specialism, such as body size, in nature. We found that when task demand varies, invariable environments select for trimodal or specialist distributions, whereas variable environments select for generalism or moderate specialism, depending on whether all tasks are usually completed. When task efficiency varies, we predict specialism unless task demand is either high or low and the environment is variable. When task importance varies task demand has much less effect and we usually predict generalism or specialism. Finally, the results show that advantage of sociality is strongest in invariable environments where most tasks are usually completed.

In previous studies, the continuous distribution of workers tends to be explained by the continuous distribution of required tasks (e.g. food size [18]), or the variation of the workers is given without any explanation. In this study, interestingly, we showed that a continuous distribution of the classes can be realized depending on the parameter values (figure 5). This means that even if the required tasks are discrete, the optimal strategy can contain a certain range of variation. Such continuous variation is very commonly observed in social animals [15–18], which can be explained by necessity of flexibility in variable environments.

Both moderate-specialism and trimodality are adaptations intermediate to specialism and generalism. They improve task performance while keeping some flexibility. While previous studies did not consider them, here we show that under stochasticity they may be expected to be common in social animals. We have found that these two types are qualitatively different and so the condition for the emergence of these types is different. A trimodal distribution has high performance during a certain range of task allocations within the colony (i.e. until all generalists allocated to either task; electronic supplementary material, figure C1a), but it shows low performance when a very biased allocation to either task is required. Contrary to this, moderate-specialism has high performance for very biased allocation, but the performance when both tasks have similar demands is not so high (electronic supplementary material, figure C1b). Because of these features, trimodal type tends to emerge when both tasks are significantly required regardless of the environmental fluctuation (i.e. figure 5b,c) or when the total task demand is relatively low with small environmental fluctuations (left-bottom area of figure 5a), while moderate-specialism tends to emerge when the fluctuation of task demand is so large that the group often face a situation where only either task is required (right area of figure 5a). The only exception is the scenario of stochasticity in importance with moderately invariable environment (upper centre area of figure 5c); then moderate-specialists are optimal because the allocation to the more important task can be advantageous.

The total task demand also has a significant impact on the optimal distribution of specialism. Importantly, in our model, group size is not explicitly considered but the task demand *per capita* of workers is considered. This means that if the increase in task demand is more rapid than the increase in workforce, the total task demand in our model increases. In other words, if a group increases the number of larvae relative to the number of workers, while the adults need food themselves, the amount of food each adult needs to collect on average would increase. This might explain the fact that with the growth of group size the distribution of worker sizes tends to be multimodal [18,37]. According to our results, the reduction of the total task demand can switch from specialism to trimodal in the scenario of task demand variability with invariable environments (right side of figure 5a) or the scenario of task efficiency variability even if the stability of environment is not changed. Since this would mean redundancy in the workforce under most conditions, this would provide an adaptive explanation for the puzzling phenomenon of large minority of workers doing little work (e.g. [38]).

We assumed that selection acts on the mean performance at the group-level under environmental variation. Of course, in many—or perhaps all—social animals fitness outcomes are not perfectly correlated. Since workers do not usually share 100% of their genes with the reproducers, there exists the potential for conflict over who reproduces. However, we took this simplification in order to derive clear predictions for the ideal case. In some systems, workers reproduce very rarely so get no direct fitness, and our predictions are likely to be accurate. This depends somewhat on whether the different tasks or degree of specialization affect direct fitness. For instance, worker honeybees (*Apis mellifera*) sometimes lay (male) eggs. If some tasks, such as foraging, have a greater mortality risk, then we might expect worker bees to be ‘reluctant’ to forage. However, if these chances of direct fitness are small, or if there is little difference in direct fitness between tasks or the degree of specialization, then this conflict over reproduction is likely to have small effects on the optimal distribution of specialism in the group.

We also showed that specialism distribution strongly depends on the types of environmental variability: whether the task demand, efficiency or importance is altered by the environment. Although in previous studies the benefit of generalists/specialists tends to be discussed only from the perspective of the trade-off of fitness in different environments, this result suggests that this trade-off cannot be simply defined because the distribution of the environment, and its influence can be variable. This implies that we need to pay further attention to details of the influence of the environment on cooperative groups. Our main aim here was to highlight the potential for environmental variation to explain some puzzling phenomena in social groups. Unfortunately, previous empirical studies rarely focused on environmental variation, especially about how the demand, efficiency and importance vary, and so the empirical data about the relationship between the environment variability and specialism are still lacking. Further empirical studies focusing on the environmental variability are required to test our hypothesis. Additionally, further developments of adaptive models of specialism in variable environments could provide a foundation which with to understand specialism in social animals.

## Data Availability

Supplementary material is available online [39].
